# Validating the Communication and Symbolic Behavior Scales–Developmental Profile Infant–Toddler Checklist (CSBS–DP ITC) Beyond Infancy in the CDKL5 Deficiency Disorder

**DOI:** 10.1007/s10803-023-06002-w

**Published:** 2023-05-15

**Authors:** Jacinta Saldaris, Helen Leonard, Kingsley Wong, Peter Jacoby, Mary Spence, Eric D. Marsh, Tim A. Benke, Scott Demarest, Jenny Downs

**Affiliations:** 1 Telethon Kids Institute, Centre for Child Health Research, The University of Western Australia, PO Box 855, West Perth, WA 6872 Australia; 2 Children’s Hospital Colorado Therapy Care, Highlands Ranch, CO USA; 3 Division of Neurology, Children’s Hospital of Philadelphia, Perelman School of Medicine, University of Pennsylvania, Philadelphia, PA USA; 4 Children’s Hospital Colorado, Paediatric Neurology, University of Colorado School of Medicine, Aurora, CO USA; 5 School of Physiotherapy and Exercise Science, Curtin University, Perth, WA Australia

**Keywords:** CDKL5 deficiency disorder, Communication, Outcome measure, Psychometric

## Abstract

CDKL5 deficiency disorder (CDD) results in early-onset epilepsy and lifelong cognitive and motor impairments. With no validated measure for communication in CDD, this study evaluated the psychometric properties of the Communication and Symbolic Behavior Scales-Developmental Profile Infant Toddler Checklist (CSBS–DP ITC). Caregivers (n = 150; affected individuals aged 1–29 years) completed the CSBS-DP ITC. Distribution of scores indicated a floor effect. There was poor divergent validity for the three-factor model but goodness of fit and convergent validity data were satisfactory for the one-factor model. Individuals with poorer overall functional abilities scored lower on the CSBS-DP ITC. Test–retest reliability was excellent. The floor effect could explain the very high reliability, suggesting problems as a sensitive outcome measure in clinical trials for CDD.

## Introduction

CDKL5 Deficiency Disorder (CDD) is classified as a developmental and epileptic encephalopathy (DEE), characterized by severe developmental impairments, epileptic seizures from infancy and other comorbidities including corticovisual impairment (CVI), poor sleep and gastrointestinal problems (Leonard et al., 2022). Currently, there are clinical trials evaluating therapeutics for CDD and capability for disease modifying therapies, such as gene therapy, is imminent (Leonard et al., 2022). A critical barrier to strong clinical trial protocols in CDD is the lack of validated outcome measures that can demonstrate meaningful interventional changes.

At a Federal Drug Administration patient-focused drug development meeting for CDD, 79% (n = 40) of caregivers identified that global developmental delay, particularly communication impairment, was one of the most burdensome symptoms for both the child and caregiver (Mingorance et al., 2020). In children with CDD aged over 1.5 years, only 16% (17/105) had attained the ability to use single words (Fehr et al., 2015) although higher proportions of children used nonverbal modes of communication (Fehr et al., 2016). Younger and older children had used similar methods of communication (Fehr et al., 2016) suggesting that developmental delays became persistent impairments.

The Communication and Symbolic Behavior Scales–Developmental Profile (CSBS–DP) is a set of assessments originally designed for screening and evaluation of communication disorders in children aged between 6 and 24 months (Wetherby & Prizant, 1993, 2002). They comprise two parent report measures, the Infant–Toddler Checklist (CSBS-DP ITC) and a Caregiver Questionnaire, and a direct observation measure, the Behavior Sample (CSBS-DP BS; Wetherby et al., 2002). The CSBS-DP ITC is a parent-report screening tool.

The CSBS–DP ITC has recently been used for children with rare genetically caused developmental encephalopathies (DEs) and DEEs. It was a secondary outcome measure in a randomized crossover trial testing mecasermin (rhIGF-1) (O'Leary et al., 2018) and is in the protocol for a phase 3 trial of trofinitide (Neul et al., 2022) in Rett syndrome. Social subscale scores were significantly higher for the treatment group in the mecasermin. For other neurogenetic syndromes (Angelman, Prader-Willi and Williams syndromes), total and subscale scores were lower than for typically developing children with the lowest scores for Angelman syndrome (Hamrick & Tonnsen, 2019) but no other evidence for its validation. In five DEEs (Dravet syndrome n = 40, KCNB1 n = 14, KCNQ2 n = 21, Lennox-Gastaut syndrome n = 36, SCN2A n = 9), raw scores correlated strongly with raw Adaptive Behavior Assessment System (ABAS) communication scale scores (Berg et al., 2022). There are limited validation data for the CSBS-DP IT in neurogenetic syndromes including DEEs.

Assessing the psychometric properties of the CSBS-DP ITC for specific populations such as CDD is necessary prior to its application in clinical trials. The aim of this study was to inform whether the CSBS-DP ITC is fit for purpose for CDD by describing the distribution of scores, evaluating the factor structure and analyzing test–retest reliability for the CSBS-DP ITC, and comparing CSBS-DP ITC scores with categorical descriptors of communication and other functional abilities.

## Methods

### Data Source

Caregivers who had completed the initial baseline questionnaire with the International CDKL5 Disorder Database (ICDD) (Fehr et al., 2015) were invited to complete an online follow up questionnaire between April 2018 and August 2019 (Leonard et al., 2021). Data were collected from 150 caregivers of children and young people with genetically confirmed CDD. Additionally, the CSBS-DP ITC was subsequently administered twice with a four-week interval to 73 caregivers. This included 30 children not in the validity dataset who had not provided follow up data in 2018 or were newly recruited.

### Measures of Functioning

#### Communication Measures

The CSBS-DP ITC comprises 24 items encompassing seven clusters which yield three composite scores: Social (comprised of the Emotion and Eye Gaze, Communication and Gestures clusters), Speech (comprised of the Sounds and Words clusters) and Symbolic (comprised of the Understanding and Object Use clusters), and a Total score (Wetherby & Prizant, 2002). Using other ICDD data collected at the same time, current communication skills were categorized as: words, gestures/signs or vocalisations, and unable.

#### Other Variables

Biological sex and genotype were described. The mutation groups were categorized according to the predicted structural/functional consequence as previously described (Fehr et al., 2015): mutations causing no functional protein, missense/in-frame mutations, truncating mutations located from aa 172 to aa 781 inclusive and truncating mutations occurring after aa 781. In those aged 12 months or older, hand function was categorized as the ability to: grasp small and large objects, pick up or hold a large but not a small object and no hand function for grasping objects. For children aged 18 months or older, mobility was categorized as: no walking, assisted and independent. Gastrostomy was categorized as: oral only, oral/gastrostomy, gastrostomy only.

Characteristics of the children and young people in the validity and reliability datasets are presented in Table 1. For the validity sample, age ranged from 1 to 29 years and 82.0% (n = 123) were female. Nearly two-thirds (n = 96) took all food orally and close to one-fifth (n = 27) communicated using some words. Approximately one third (n = 57, 38%) could grasp and pick up a selection of large and small objects and 33 (22.3%) could walk independently. Each of the four mutation groups (Fehr et al., 2015) were represented. The reliability sample had similar distributions for biological sex, genotype, walking and communication, but higher proportions took all food orally (74%) and could grasp and pick up a selection of large and small objects (51%) (Table 1).

**Table 1:** Characteristics of individuals with CDD in the CFA Analysis and Reliability Analysis, and CSBS-DP ITC total score data for questionnaires with no missing data

Variable	CFA analysis	Reliability analysis	CSBS-DP ITC total scores^a^		
			n (%)	Median (IQR)	% items scored 0 Mean (SD)
Total participants	n = 150	n = 73	n = 131	12 (5,18)	57.4 (25.3)
Age group, n (%)					
1 < 3 years	17 (11.3)	17 (23.3)	14 (10.7)	9(5,22)	58.0 (26.2)
3 < 7 years	40 (26.7)	19 (26.0)	37 (28.2)	8 (4,15)	64.9 (22.6)
7–12 years	50 (33.3)	11 (15.1)	48 (36.6)	14 (6,19.5)	52.7 (26.5)
> 12 years	43 (28.7)	26 (35.6)	32 (24.4)	11.5 (6.5,20.5)	55.6 (25.0)
Median age (IQR, min–max), years	9.1 (5.0–13.9, 1–29.0)	7.7 (3.3–14.6,1–24.4)	–	–	–
Gender, n (%)					
Female	123 (82.0)	62 (84.9)	108 (82.4)	12.5 (6,19)	55.7 (25.0)
Male	27 (18.0)	11 (15.1)	23 (17.6)	7 (4,15)	65.4 (25.3)
Genotype, n (%)					
No functional protein	39 (26.0)	18 (24.7)	34 (26.0)	11 (6,17)	57.6 (26.5)
Missense/in-frame mutation within catalytic domain	40 (26.7)	25 (34.2)	34 (26.0)	11 (5,17)	60.0 (24.8)
Truncation from aa 172 to aa 781	42 (28.0)	17 (23.3)	36 (27.5)	9 (5.5,15)	62.5 (22.0)
Truncation after aa 781	21 (14.0)	9 (12.3)	19 (14.5)	18 (7,33)	48.2 (25.1)
Not grouped	8 (5.3)	4 (5.5)	8 (6.1)	19 (12.5,33.5)	44.3 (31.6)
Ability to walk, n (%) (n = 148)					
Independent	33 (22.3)	15 (20.5)	32 (24.4)	27 (18,41.5)	29.8 (22.2)
Assisted	16 (10.8)	9 (12.3)	13 (9.9)	13 (6,18)	62.2 (19.6)
Unable	99 (66.9)	49 (67.1)	86 (65.6)	7 (5,14)	73.0 (16.8)
Communication, n (%)					
Words	27 (18.0)	10 (13.7)	26 (19.8)	28.5 (20,46)	26.6 (23.4)
Gestures, signs or vocalisations	93 (62.0)	52 (71.2)	79 (60.3)	11 (6,15)	61.4 (19.5)
Unable	30 (20.0)	11 (15.1)	26 (19.8)	5 (3,9)	76.0 (13.8)
Feeding patterns, n (%)					
Oral only	96 (64.0)	54 (74.0)	84 (64.1)	15 (7,24.5)	49.8 (25.0)
Oral and enteral	21 (14.0)	10 (13.7)	21 (16.0)	10 (3,15)	66.1 (24.2)
Enteral only	33 (22.0)	9 (12.3)	26 (19.8)	5 (3,9)	75.2 (13.5)
Hand function, n (%)					
Grasp any object	57 (38.0)	37 (50.7)	52 (39.7)	19 (14,34)	38.5 (24.2)
Hold or grasp large object only	34 (22.7)	21 (28.8)	29 (22.1)	12 (6,15)	62.6 (19.0)
No grasping	59 (39.3)	15 (20.5)	50 (38.2)	6 (4,9)	74.0 (13.9)

n, number of individuals

Parentheses next to n indicate % values or IQR and range values as indicated next to the first level variable label

^a^Questionnaires with no missing items

### Statistical Analysis

Descriptive statistics were used to summarize the characteristics of the individuals with CDD as well as their CSBS-DP ITC item and total scores. Confirmatory factor analysis was performed to verify the factor structure of the composite (social, speech and symbolic) scores. The three factors were allowed to be correlated with each other, but no correlated errors were permitted. All items were treated as ordinal variables and hence the weighted least square mean and variance adjusted (WLSMV) estimator was used (Lei & Shiverdecker, 2020). Standardized factor loadings were reported. Goodness of fit of the 3-factor model was assessed using the following statistics: CMIN/df value, root mean square error approximation (RMSEA), the Comparative Fit Index (CFI) and the Tucker–Lewis Index (TLI). Cronbach’s alpha, the composite reliability and average variance extracted (AVE) statistics were calculated for each factor to assess convergent validity. The maximum correlation squared (MCS) value was calculated for each factor and compared with AVE to assess divergent validity. As the divergent validity of the 3-factor model was found to be sub-optimal, confirmatory factor analysis of 1-factor model was performed and associated goodness of fit statistics were obtained. The relationships between the CSBS-DP ITC total score as continuous variable and functional abilities (walking, communication, feeding method, hand function) were investigated using simple linear regression. Multivariable regression was also carried out to examine the associations adjusted for age and genotype. Point estimates and their 95% confidence intervals were reported. To avoid unnecessary burden on the families we recruited a smaller number for the test–retest reliability analysis whilst still retaining good precision for the reliability estimate. With 73 families, we calculated that the 95% confidence interval around our anticipated reliability value of ICC = 0.9 (good agreement) would lie between 0.85 and 0.95. Test–retest reliability was assessed using intra-class correlations (ICCs). ICCs were interpreted as ≤ 0.40 slight agreement, 0.41–0.60 fair agreement, 0.61–0.80 moderate agreement, and 0.81–1.00 substantial agreement (Landis & Koch, 1977). Statistical analysis of data was performed using Stata 15.1 (StataCorp. 2017. Stata Statistical Software: Release 15. College Station, TX: StataCorp LLC.), and, for confirmatory factor analysis, Mplus version 8.5 (Muthén & Muthén, 1998–2017).

## Results

The mean (SD) CSBS-DP total score was 14.6 (12.7) and the median (IQR) was 12 (5–18) out of a total possible score of 57, based on a complete dataset for 131 individuals (Table 1). The percent of items with a score of 0 is shown in Table 1. For all participants, over half (57.4%) of items received a score of 0. There was a greater percentage of items that scored 0 (65.4%) for males compared to females (55.7%). Individuals with lower abilities in walking, communication, and hand function had in a greater percentage of items scored 0. Also, individuals who are fed through GI tube only had a higher percent of 0 scores (75.2%) compared to children who engaged in oral feeding only (5.02%).

**Fig. 1:**
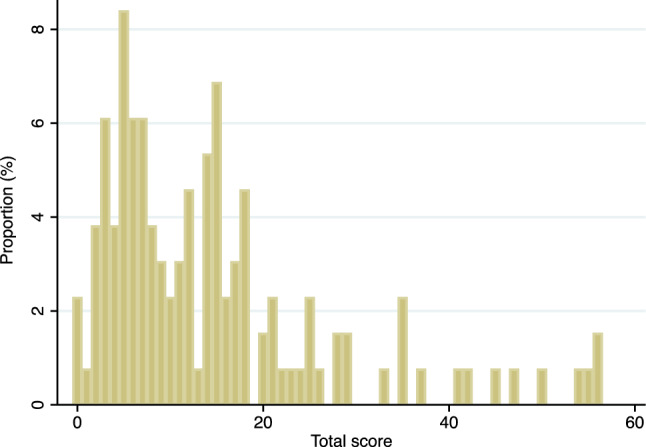
Frequency distribution of CSBS DP Infant–Toddler checklist total score

For each item, each response category was scored by at least one individual (Table 2) although the total score was highly skewed (1.6; Fig. 1), indicating a floor effect. The majority scored zero for most items, but seven items contributed greater variability to the scores: item 1 (Do you know when your child is happy and when your child is upset?), item 3 (Does your child smile or laugh while looking at you?), item 6 (When you are not paying attention to your child, does he/she try to get your attention?), item 14 (Does your child use sounds or words to get attention or help?), item 16 (About how many of the following consonant sounds does your child use: ma, na, ba, da, ga, wa, la, ya, sa, sha?), item 19 (When you call your child’s name, does he/she respond by looking or turning toward you?) and item 20 (About how many different words or phrases does your child under- stand without gestures?) (Table 2).

**Table 2:** Descriptive statistics of CSBS DP Infant–Toddler checklist item scores, including composite (e.g., SOCIAL), cluster (e.g., Emotion and Eye Gaze), and total scores, in 150 individuals with CDD

Composite/cluster/item	n	Mean (SD)	Median (IQR)	Range	Number of points scored (for checklist items only), n (%)					
					0	1	2	3	4	Missing
SOCIAL^a^	142	7.0 (6.0)	5 (3,10)	0–26						
Emotion and Eye Gaze (Items 1–4)	146	3.6 (1.9)	3 (2,5)	0–8						
1. Know when child is happy/upset	150	1.8 (0.5)	2 (2,2)	0–2	3 (2.0)	28 (18.7)	119 (79.3)	–	–	0
2. When playing child checks to see if you are watching	149	0.3 (0.6)	0 (0,1)	0–2	111 (74.0)	25 (16.7)	13 (8.7)	–	–	1 (0.7)
3. Smile/laugh while looking at you	149	1.1 (0.7)	1 (1,2)	0–2	34 (22.7)	69 (46.0)	46 (30.7)	–	–	1 (0.7)
4. Child looks when you point	148	0.4 (0.6)	0 (0,1)	0–2	110 (73.3)	24 (16.0)	14 (9.3)	–	–	2 (1.3)
Communication (Items 5–8)	146	2.1 (2.2)	1 (0,3)	0–8						
5. Let you know when needs help to reach toy	149	0.6 (0.8)	0 (0,1)	0–2	87 (58.0)	35 (23.3)	27 (18.0)	–	–	1 (0.7)
6. Tries to get your attention	148	0.9 (0.8)	1 (0,2)	0–2	55 (36.7)	51 (34.0)	42 (28.0)	–	–	2 (1.3)
7. Do things to make you laugh	150	0.4 (0.6)	0 (0,1)	0–2	110 (73.3)	27 (18.0)	13 (8.7)	–	–	0
8. Try to get you to notice things	149	0.2 (0.5)	0 (0,0)	0–2	131 (87.3)	10 (6.7)	8 (5.3)	–	–	1 (0.7)
Gestures (Items 9–13)	149	1.3 (2.4)	0 (0,1)	0–10						
9. Pick up objects and give to you	149	0.3 (0.6)	0 (0,0)	0–2	119 (79.3)	15 (10.0)	15 (10.0)			1 (0.7)
10. Show objects with giving	149	0.2 (0.6)	0 (0,0)	0–2	125 (83.3)	14 (9.3)	10 (6.7)			1 (0.7)
11. Wave to greet people	149	0.2 (0.6)	0 (0,0)	0–2	123 (82.0)	15 (10.0)	11 (7.3)			1 (0.7)
12. Point to objects	149	0.2 (0.5)	0 (0,0)	0–2	130 (86.7)	10 (6.7)	9 (6.0)			1 (0.7)
13. Nod head to indicate yes	149	0.3 (0.6)	0 (0,1)	0–2	112 (74.7)	27 (18.0)	10 (6.7)			1 (0.7)
SPEECH^b^	148	3.3 (3.6)	2 (1,5)	0–14						
Sounds (Items 14–16)	148	2.6 (2.4)	2 (1,4)	0–8						
14. Use sounds/words to get help	149	1.0 (0.8)	1 (0,2)	0–2	47 (31.3)	57 (38.0)	45 (30.0)	–	–	1 (0.7)
15. String sounds together	149	0.5 (0.7)	0 (0,1)	0–2	96 (64.0)	31 (20.7)	22 (14.7)	–	–	1 (0.7)
16. Consonant sounds used	148	1.1 (1.3)	1 (0,2)	0–4	63 (42.0)	39 (26.0)	22 (14.7)	13 (8.7)	11 (7.3)	2 (1.3)
Words (Items 17–18)	149	0.7 (1.5)	0 (0,1)	0–6						
17. Words used meaningfully that you recognise	149	0.5 (1.0)	0 (0,1)	0–4	107 (71.3)	25 (16.7)	5 (3.3)	5 (3.3)	7 (4.7)	1 (0.7)
18. Put two words together	149	0.1 (0.5)	0 (0,0)	0–2	134 (89.3)	8 (5.3)	7 (4.7)	–	–	1 (0.7)
SYMBOLIC^c^	137	4.0 (3.8)	3 (1,5)	0–17						
Understanding (Items 19–20)	143	2.2 (1.8)	2 (1,4)	0–6						
19. Child responds to their name	144	1.0 (0.7)	1 (0,2)	0–2	37 (24.7)	69 (46.0)	38 (25.3)	–	–	6 (4.0)
20. Child understands words/phrase without gestures	148	1.2 (1.3)	1 (0,2)	0–4	63 (42.0)	34 (22.7)	28 (18.7)	9 (6.0)	14 (9.3)	2 (1.3)
Object Use (Items 21–24)	143	1.9 (2.5)	1 (0,3)	0–11						
21. Shows interest playing with a variety of objects	148	0.7 (0.8)	0 (0,1)	0–2	79 (52.7)	40 (26.7)	29 (19.3)	–	–	2 (1.3)
22. Objects used appropriately	149	0.8 (1.1)	0 (0,1)	0–4	77 (51.3)	43 (28.7)	12 (8.0)	10 (6.7)	7 (4.7)	1 (0.7)
23. Child can stack blocks	146	0.2 (0.7)	0 (0,0)	0–3	130 (86.7)	7 (4.7)	1 (0.7)	8 (5.3)	–	4 (2.7)
24. Child pretends to play with toys	147	0.1 (0.4)	0 (0,0)	0–2	134 (89.3)	8 (5.3)	5 (3.3)	–	–	3 (2.0)
TOTAL^d^	131	14.6 (12.7)	12 (5,18)	0–56						

n, number of individuals; SD, standard deviation; IQR, interquartile range

^a^Skewness 1.6, kurtosis 5.0

^b^Skewness 1.5, kurtosis 4.6

^c^Skewness 1.6, kurtosis 5.5

^d^Skewness 1.6, kurtosis 5.2

### Confirmatory Factor Analysis

Confirmatory factor analysis was conducted for 150 individuals.

#### Three-Factor Analysis

Factor loadings for each of the items in the three-factor model were all greater than 0.6 (Table 3). The three-factor model showed satisfactory indices of relative fit using CMIN/df (1.387), RMSEA (0.051), CFI (0.991) and TLI (0.990; Table 4). Cronbach’s alpha values for the three-factor model ranged from 0.850 for Symbolic to 0.924 for Social, composite reliability values ranged from 0.934 for Symbolic to 0.975 for Social, and average variance extracted ranged from 0.708 for Symbolic to 0.820 for Speech, indicating satisfactory convergent reliability (Table 4). However, for each domain, the AVE values were smaller than the MCS values, therefore giving clear evidence of a lack of divergent validity of these domains (Hu & Bentler, 1999).

**Table 3:** Standardized factor loading (95% confidence interval) values from 3-factor and 1-factor confirmatory factor analysis for 150 individuals with CDD

Composite/cluster/item	3-Factor model			1-Factor model
	Factor 1 (Social)	Factor 2 (Speech)	Factor 3 (Symbolic)	
**Social**				
*Emotion and Eye Gaze*				
1. Know when child is happy/upset	0.89			0.88
2. When playing child checks to see if you are watching	0.80			0.79
3. Smile/laugh while looking at you	0.71			0.70
4. Child looks when you point	0.92			0.92
*Communication*				
5. Let you know when needs help to reach toy	0.80			0.79
6. Tries to get your attention	0.85			0.84
7. Do things to make you laugh	0.91			0.90
8. Try to get you to notice things	0.97			0.97
*Gestures*				
9. Pick up objects and give to you	0.95			0.95
10. Show objects with giving	0.92			0.92
11. Wave to greet people	0.88			0.87
12. Point to objects	0.98			0.97
13. Nod head to indicate yes	0.58			0.57
**Speech**				
*Sounds*				
14. Use sounds/words to get help		0.75		0.70
15. String sounds together		0.91		0.88
16. Consonant sounds used		0.89		0.86
*Words*				
17. Words used meaningfully that you recognise		0.95		0.93
18. Put two words together		1.00		0.97
**Symbolic**				
*Understanding*				
19. Child responds to their name			0.73	0.73
20. Child understands words/phrase without gestures			0.81	0.81
*Object use*				
21. Shows interest playing with a variety of objects			0.65	0.65
22. Objects used appropriately			0.83	0.83
23. Child can stack blocks			1.00	1.00
24. Child pretends to play with toys			0.97	0.97

**Table 4:** Goodness of fit statistics, internal consistency and discriminant validity statistics from the 3-factor and 1-factor confirmatory factor analysis

Goodness of fit^a^	3-Factor model	1-Factor model
CMIN/df	1.408	1.545
Root Mean Square Error of Approximation	0.052	0.061
Comparative Fit Index	0.991	0.988
Tucker-Lewis Index	0.990	0.986

Validity	Factor 1 (Social)	Factor 2 (Speech)	Factor 3 (Symbolic)	1-Factor model
Cronbach’s alpha^b^	0.924	0.880	0.850	0.959
Composite reliability^b^	0.975	0.958	0.934	0.985
Average variance extracted^c,d^	0.750	0.820	0.708	0.733
Maximum correlation squared^d^	0.994	0.876	0.994	N/A

^a^Commonly used rules for satisfactory goodness of fit are (Hu & Bentler, 1999). 1) CMIN/df is < 3; 2) the Root Mean Squared Error of Approximation is < 0.08; and 3) the Comparative Fit Index and the Tucker-Lewis Index are at least 0.9

^b^Satisfactory internal consistency indicated if Cronbach’s alpha and composite reliability scores > 0.7. (Hu & Bentler, 1999)

^c^Satisfactory AVE is > 0.5. (Hu & Bentler, 1999)

^d^Satisfactory divergent validity indicated if the average variance extracted value is > the maximum correlation squared value. (Hu & Bentler, 1999)

#### One-Factor Analysis

Factor loadings for each of the items in the single factor model were all greater than 0.6 (Table 3). This model showed satisfactory indices of relative fit using CMIN/df (1.542), RMSEA (0.060), CFI (0.988) and TLI (0.987; Table 4). For the one-factor model, internal consistency values were extremely high (Cronbach’s alpha 0.959, composite reliability 0.985) and convergent validity was satisfactory (AVE 0.733) (Table 4).

### Comparison of Known Groups

Comparisons of the CSBS-DP ITC one-factor score for the functional group categories using data from 131 individuals is shown in Table 5. This analysis was limited to individuals aged 18 months or older at the time of questionnaire completion to avoid evaluating skills, such as walking, in children who would not yet be expected to. Children who required assistance (coefficient − 16.9, 95% CI − 22.9, − 10.8) or were unable to walk (coefficient − 20.5, 95% CI − 24.3, − 16.6) had significantly lower scores than those who walked independently. Children who had oral and enteral feeding (coefficient − 6.9, 95% CI − 12.7, − 1.2) or enteral feeding only (coefficient − 10.9, 95% CI − 16.2, − 5.6) had significantly lower scores than oral feeding only, also adjusting for age-group and genotype. Children able to grasp large objects only (coefficient − 12.4, 95% CI − 17.2, − 7.7) or had no grasping ability (coefficient − 16.7, 95% CI − 20.7, − 12.7) had significantly lower scores than children able to grasp small and large objects. For all functional abilities, those in the lower performing group scored lower on the CSBS-DP ITC.

**Table 5:** Univariable linear regression analysis of the associations between the CSBS-DP ITC total score and covariates (n = 131)

Covariates	Functional categories	n (%)	Univariable	
			β (95% CI), *P* value	Predicted mean (95% CI)
Communication	Words	25 (19.1)	Ref	31.3 (27.5, 35.1)
	Gestures, signs or vocalisations	80 (61.1)	− 19.3 (− 23.7, − 15.0), < 0.01	12.0 (9.9, 14.1)
	Unable	26 (19.9)	− 24.6 (− 29.9, − 19.3), < 0.01	6.7 (3.0, 10.4)
Ability to walk^a^	Independent	32 (24.8)	Ref	29.8 (26.6, 33.1)
	Assisted	13 (10.1)	− 16.9 (− 22.9, − 10.8), < 0.01	12.9 (7.8, 18.0)
	Unable	84 (65.1)	− 20.5 (− 24.3, − 16.6), < 0.01	9.3 (7.3, 11.4)
Feeding patterns	Oral only	84 (64.1)	Ref	17.9 (15.3, 20.5)
	Oral and enteral	21 (16.0)	− 6.9 (− 12.7, − 1.2), 0.02	11.0 (5.8, 16.1)
	Enteral only	26 (19.9)	− 10.9 (− 16.2, − 5.6), < 0.01	7 (2.4, 11.6)
Hand function	Grasp any object	51 (38.9)	Ref	23.9 (21.0, 26.7)
	Grasp large object only	29 (22.1)	− 12.4 (− 17.2, − 7.7), < 0.01	11.4 (7.6, 15.2)
	No grasping	51 (38.9)	− 16.7 (− 20.7, − 12.7), < 0.01	7.2 (4.3, 10.0)

^a^Limited to individuals aged 18 months or older at the time of questionnaire completion (n = 129); n, number of individuals; β, beta coefficient; CI, confidence interval; Ref, reference category

### Test–retest Reliability

Test–retest reliability had an extremely high level of agreement (ICC = 0.96, 95% CI 0.94–0.98). The mean interval between test and retest was 36.6 days.

## Discussion

This is the first study to examine the distribution of scores, validity and reliability of the CSBS-DP ITC in a large sample of children and young adults with CDD, an essential task as no communication measures have been validated for CDD. This work is critical if clinical trials with non-seizure endpoints are to successfully proceed to a reliable outcome. This validation study provided mixed evidence of the suitability of the CSBS-DP ITC for the CDD population. Although the three-factor model did not show satisfactory divergent validity, the one-factor model showed satisfactory convergent and known groups validity. However, the distribution of scores showed a floor effect, and the internal consistency and test–retest reliability values were unusually high.

There is increasing evidence describing the psychometric properties of the CSBS-DP ITC in neurogenetic syndromes including DEEs, beyond its original evaluations as an early childhood screening tool for autism (Wetherby et al., 2004). When used as an outcome measure in clinical trials for RTT, significant differences were observed for the social composite score (O'Leary et al., 2018). This prompted us to test the CSBS-DP ITC with CDD. More recent evidence suggests that CSBS-DP ITC scores correlate with other communication measures for a group of DEEs (Berg et al., 2022) and scores differ by diagnostic group as would be expected. Consistently in our study, scores differed by broad functional ability groups. We used CFA to investigate psychometric properties further and reject the three-factor model for CDD because the factors were too highly correlated to justify separation of the items into the three domains. The one-factor model showed more satisfactory psychometric properties.

However, the distribution of the CSBS-DP ITC total score was very right skewed, indicating a floor effect, meaning the CSBS-DP ITC may be problematic for use in the CDD population and is dependent on the meaningfulness of change in a clinical trial setting for which data are not yet available. CDD is associated with severe impairments and there is high risk of a floor effect when measuring functional abilities with scales that were designed to evaluate a larger range of skills and pay limited attention to small incremental skills at the lower range of functioning (Berg et al., 2020). For example, approximately 75% of items were scored as 0 for groups with the most severe impairments. We note that the floor effect appears to have been less marked for other DEEs (Berg et al., 2022). We also observed an extremely high level of agreement with test–retest reliability, with minimal within subject noise. This could be related to the floor effect and the CSBS having poor granularity in the subtle communication skills associated with severe disability.

Other aspects of the CSBS-DP ITC could be advantageous. It includes questions on social communication skills, as does the Communication Matrix (Rowland & Fried-Oken, 2010), consistent with the notion that social communication is an important part of quality of life (Tangarorang et al., 2019). In addition, rather than scoring as “Never” or “0”, the CSBS-DP ITC uses the wording “not yet”. Parents participating in our Consumer Reference Group meetings have described how they value the scoring category "not yet", especially when their child achieves few skills. Parents with a child with CDD live with day-to-day challenges to their mental health (Mori et al., 2017) and measurement should be efficient and strengths-based to reduce their burden. On the other hand, it is also important to note the limitations of the CSBS-DP ITC. For instance, item 1 asks if the child indicates being happy or upset combining two concepts within the one item. Potential use of an Augmentative and Alternative Communication (AAC) device is not addressed and it can be difficult for caregivers to know exactly how many words or phrases some children can understand, if the child has extremely limited expressive communication and movement skills, and is without access to an AAC device (Mingorance et al., 2020). Supplementing the CSBS-DP ITC with additional items could reduce the floor effect and enable greater capacity for the measure to identify responsiveness to change if impairments are severe. Qualitative studies to understand communication in children with severe impairments and mapping of items across available communication measures would inform potential modifications.

The ICDD was used for recruitment, resulting in a sample size well powered for this analysis. With 24 items in the checklist, the sample was larger than the generally recommended sample size of five participants per item for factor analysis (Streiner, 1994). Parent reported questionnaires are commonly used in pediatric and intellectual disability fields and there is evidence for their suitability in young children and those with intellectual disabilities (Fisher et al., 2014; Wetherby et al., 2002), suggesting suitability for CDD. Our findings could be relevant to other DEE conditions with severe communication impairments although additional validation studies are needed. We acknowledge that further validation studies of other available communication outcome measures could yield suitable measures of communication for DEE conditions.

## Conclusions

This study is the first to generate validation data for a communication measure for CDD. Our mixed findings suggest caution in using the CSBS-DP ITC in clinical trials. We found evidence that using the total score is preferable to the three composite scores but that there could be value in exploring whether additional items could buttress its capacity to describe communication more incrementally at the most impaired levels. There is an urgent need for validated outcome measures for CDD and other DEEs to support upcoming clinical trials of novel treatments, especially with targeted gene therapies on the horizon. Our study represents affirmative action in this arena.
